# The Application of Combined PET/MRI in Staging and Response Assessment of Rectal Cancer

**DOI:** 10.3390/jcm14207436

**Published:** 2025-10-21

**Authors:** Elima Hussain, Vipul R. Sheth

**Affiliations:** Department of Radiology, Stanford University, Stanford, CA 94305, USA; elimah@stanford.edu

**Keywords:** PET/MRI, rectal cancer, colorectal cancer, hybrid imaging, staging, response assessment, radiomics

## Abstract

**Background/Objectives:** Rectal cancer is a significant global health concern, requiring precise staging and response assessment to make treatment decisions. Magnetic resonance imaging (MRI) is the standard imaging modality for evaluating tumor stage and treatment response. Positron emission tomography/computed tomography (PET/CT) offers complementary insights into pelvic lymph node involvement, tumor response, and distant metastases. Integrating PET and MRI into a hybrid PET/MRI modality can provide superior assessment of tumor staging and response compared to conventional imaging techniques. This review shares an update on the role of PET/MRI in rectal cancer staging and treatment response assessment. **Methods:** A systematic review of the current literature was conducted by two independent reviewers. This study utilized databases including Embase, Biosis, PubMed, Scopus, and Web of Science, employing the following keywords as eligibility criteria: “PET/MRI” OR “PET/MR” AND “rectal cancer” OR “colorectal cancer” AND “staging” AND “treatment assessment” OR “planning”. The inclusion criteria were that studies must examine cancer staging and response assessment. The exclusion criteria for the search were letters to the editors, abstracts, and case reports; studies that included fewer than five patients; studies that included cancer other than rectal or colorectal cancer; studies that did not utilize PET/MRI for rectal cancer staging and assessment; and non-human studies. **Results:** PET/MRI demonstrates potential advantages over conventional imaging, providing superior soft tissue contrast, functional imaging capabilities, and improved lesion characterization. A total of ten studies suggest that PET/MRI may enhance tumor staging accuracy and better assess pelvic lymph node involvement than PET/CT and MRI alone; in four studies, PET/MRI also showed higher response accuracy. Challenges remain in standardizing imaging protocols, validating PET tracers, and encouraging widespread clinical adoption. **Conclusions**: PET/MRI has the potential to offer a superior imaging solution for rectal cancer staging and treatment response assessment. While preliminary studies highlight its advantages over PET/CT and MRI alone, further research is needed to establish standardized protocols, validate PET tracers for routine clinical use, and improve imaging quality through attenuation and motion correction.

## 1. Introduction

Rectal cancer is a significant global health concern, accounting for 30–40% of colorectal cancers and increasing in incidence. According to GLOBOCAN 2020 estimates, colorectal cancer is the third most commonly diagnosed cancer globally, with over 1.9 million new cases and approximately 935,000 deaths reported in 2020 [1]. Accurate staging and response assessment are crucial for guiding treatment decisions and managing patient prognosis in rectal cancer.

The various imaging modalities available include endoanal ultrasound (EUS), multidetector computed tomography (MDCT), magnetic resonance imaging (MRI), positron emission tomography (PET), and PET/CT [2,3,4]. MRI is the recommended modality for initial tumor staging and assessment of the mesorectum, mesorectal fascia, and regional lymph nodes, and for subsequent response assessment due to excellent soft tissue contrast [5]. The endoanal ultrasound (EUS) modality can be used for tumor (T) staging in early stages to distinguish between T1 and T2 tumors; however, it is limited by operator dependency, reduced accuracy in cases of bulky or advanced disease, and an inability to evaluate stenotic lesions. Computerized tomography (CT) of the chest, abdomen, and pelvis is essential for assessing distant metastases. CT is suboptimal for assessing tumor invasion depth and the mesorectal fascia. Although malignant lymph nodes can be detected using EUS, CT, and MRI based on size, morphology, and internal characteristics, these techniques have limitations, particularly in terms of specificity following neoadjuvant treatments [6].

The use of PET/CT in rectal cancer imaging typically involves [^18^F]Fluorodeoxyglucose, which has a picomolar functional resolution and a clear role in lymph node staging, evaluation of distant metastases, and follow-up [7,8]. PET/CT may be useful for lymph node assessment, detecting suspected disease recurrence, and identifying occult metastases in cases of suspected systemic disease. A comprehensive review by Nicastri et al. [9] discussed the clinical significance of occult lymph node metastasis using the immunohistochemistry technique in colorectal cancer patients. However, evidence on the clinical value of PET/CT and MRI regarding evaluating lymph node metastases in rectal cancer remains limited. Given this gap, this review aims to summarize the current findings and highlight areas requiring further research in the clinical assessment of lymph node metastases for staging rectal cancer patients.

The [^18^F]FDG-PET/MRI hybrid modality combines the strengths of PET and MRI, offering high-resolution anatomical imaging with functional techniques such as diffusion-weighted imaging (DWI), T1/T2 mapping, and dynamic contrast-enhanced (DCE) imaging [10,11]. Advancements in [^18^F]FDG-PET/MRI protocols, including reduced scan times and improved motion correction, have further enhanced its clinical utility in rectal cancer, as highlighted by Nensa et al. [12]. There are studies indicating that [^18^F]FDG-PET/MRI surpasses [^18^F]FDG-PET/CT and CT in detecting liver metastases [13], a critical factor in treatment planning. Emerging evidence also suggests that [^18^F]FDG-PET/MRI is more effective than MRI alone in detecting residual disease after total neoadjuvant therapy [14]. This is particularly relevant for patients being considered for rectum-sparing treatment strategies, such as a “watch-and-wait” approach [15]. All these studies underscore the potential of [^18^F]FDG-PET/MRI to prevent overtreatment or unnecessary surgeries in such patients with a complete or near-complete response. This review provides an overview of [^18^F]FDG-PET/MRI’s role in rectal cancer staging, treatment response assessment, and its potential to guide rectum-sparing strategies.

This review paper is organized as follows: Section 2 describes the materials and methods used for the selective literature search; and Section 3 describes the results, including the following sub-categories of the study design: technical aspects of [^18^F]FDG-PET/MRI; the role of PET/MRI in TNM staging targeted for rectal cancer; the role of PET/MRI in evaluating responses to neoadjuvant therapy or chemoradiation therapy; and the emerging and existing novel PET tracers for diagnosis and monitoring.

## 2. Materials and Methods

**Figure 1:**
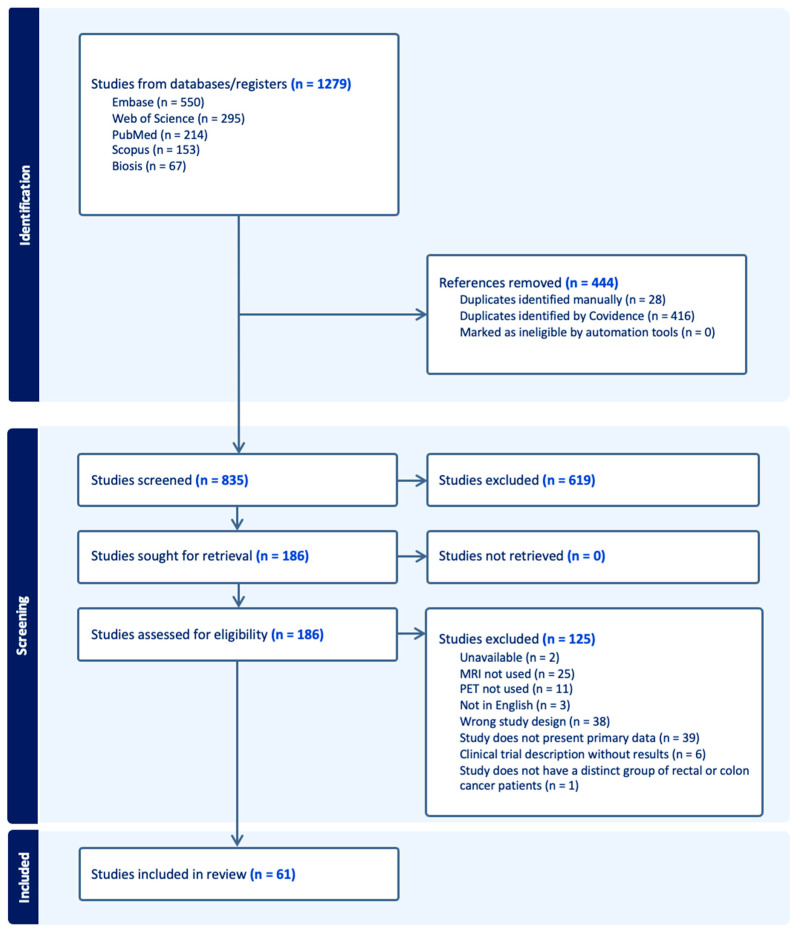
PRISMA flow diagram for literature search selection.

A systematic literature search was conducted following the standard Preferred Reporting Items for Systematic Reviews and Meta-Analyses (PRISMA) guidelines [16] (Figure 1). The comprehensive search was made following the Embase, Biosis, PubMed, Scopus, and Web of Science databases using the keywords and query “PET/MRI” OR “PET/MR” AND “rectal cancer” OR “colorectal cancer” AND “staging” AND “treatment assessment” OR “planning”. The inclusion criteria were all studies related to rectal or colorectal cancer staging and response assessment, and the exclusion criteria for the search were (1) letters to the editor, abstracts, and case reports; (2) studies that included less than five patients; (3) studies that included cancer other than rectal or colorectal cancer; (4) studies that did not utilize PET/MRI for rectal cancer staging and assessment; and (5) non-human studies. The literature search was further categorized into technical aspects of PET/MRI, scope of PET/MRI in rectal cancer staging, PET/MRI for response assessment for rectal cancer, and emerging PET tracers in rectal cancer. Study selection was conducted by two people or reviewers who independently screened the titles, abstracts, and full-text articles using the Covidence systematic review software (Veritas Health Innovation, Melbourne, Australia. Available at www.covidence.org). The differences in screening opinions between the reviewers were discussed and any disagreements were resolved through discussion when necessary. Standardized PICO (T) structure for ideal intervention reviews and risk of bias was followed as recommended by the Cochrane tool, which is supported by Covidence. The Covidence software also calculates the Kappa score, which was reported as 85% for title and abstract screening and 78% for full-text reviews.

## 3. Results

### 3.1. Technical Aspects of FDG-PET/MRI

PET scanners detect gamma ray photons emitted from radiotracers, while MRI uses magnetic fields and radiofrequency waves to generate detailed soft tissue contrast and functional information. The development of [^18^F]FDG-PET/MRI scanners has addressed some key challenges, such as the incompatibility of traditional PET photomultiplier tubes with MRI’s strong magnetic fields and rapidly changing gradient fields. It also focuses on maintaining MRI performance and creating methods which are template/atlas-based, segmentation-based, and reconstruction-based for attenuation correction [17,18,19]. An active area of research is the development of MRI-based motion correction strategies that help minimize misalignment artifacts [20]. There are two commercially available PET/MRI systems, the Biograph mMR (Siemens Healthcare GmbH, Erlangen, Germany) and the Signa PET/MRI (GE Healthcare, Waukesha, WI, USA), and both systems have some technological differences contributing to variations in spatial resolution, sensitivity, and overall image quality, but this is beyond the scope of this review.

[^18^F]FDG-PET/CT is widely available as an established imaging protocol with proven clinical potentials in inflammation, cardiac, and neurological imaging [19,20]. It offers quantitative accuracy and rapid scan times but is limited by poor soft tissue contrast and ionizing radiation. [^18^F]FDG-PET/MRI provides superior soft tissue contrast with the application of diffusion-weighted imaging (DWI). It allows better motion correction and eliminates patient radiation exposure. However, its adoption is hindered by limited availability, longer scan times, evolving protocols, and the need for specialized technologists. [^18^F]FDG-PET/MRI may be a preferred option for younger patients and those requiring repeated imaging due to reduced radiation exposure. Studies have shown [^18^F]FDG-PET/MRI’s high diagnostic performance in cancers such as neuroendocrine tumors, prostate cancer, gynecologic malignancies, breast cancer, and lymphoma [21]. However, its superiority over [^18^F]FDG-PET/CT still remains uncertain due to limited studies with small patient cohorts and heterogeneous endpoints [22]. Therefore, future research should focus on identifying specific clinical applications and optimizing the MRI sequences for PET integration. This could enhance its clinical benefits in cancer imaging while addressing scan time limitations. The widespread clinical adoption of [^18^F]FDG-PET/MRI remains challenging due to high operational costs, technical complexities, and the need for robust clinical validation.

### 3.2. MRI Sequences in Rectal Cancer Imaging

Many studies highlight high-spatial-resolution T2-weighted imaging as the most important MRI sequence in the evaluation of rectal cancer and related anatomic structures. The standard rectal MRI protocol for evaluating rectal cancer typically involves two-dimensional (2D) fast spin echo (FSE) T2-weighted sequences without fat suppression using a high-resolution and small field of view with a slice thickness of less than 3 mm [23]. Images should be acquired in the oblique axial plane (perpendicular to the tumor), sagittal plane (following the tumor’s longitudinal axis), and oblique coronal plane (parallel to the anal canal) to accurately assess tumor invasion in relation to the muscularis propria and anal sphincter. These sequences have demonstrated high diagnostic accuracy (90–100%) in evaluating tumor spread to the mesorectal fascia (MRF) and surrounding organs. FSE T2-weighted MRI, with a larger field of view in the axial plane, allows for the assessment of distant lymph node chains, while the sagittal plane provides precise tumor localization, height measurement, and analysis of its relationship to midline structures like the anal verge. These protocols have been recommended by the MERCURY group for their effectiveness in rectal cancer staging [24]. There is still debate in some studies highlighting certain protocols not recommended for malignant rectal tumor assessment. Hoeffel et al. [25] have indicated that certain imaging protocols should not be routinely used in rectal MR imaging. Their study recommended against the use of air insufflation for rectal distension with contrast material. T2-weighted imaging with fat suppression is also not recommended as part of the standard protocol. High b-value diffusion-weighted imaging (DWI) (≥800 sec/mm^2^) can improve staging accuracy, especially for restaging after chemoradiotherapy and for enhancing the detection of tumors and lymph nodes during primary staging [26]. Usage of spasmolytic agents like glucagon or hyoscine butyl bromide may help reduce motion artifacts from peristalsis if or when given before the MR exam or before exhibiting motion-sensitive sequences such as DWI or DCE [20]. Griethuysen et al. [27] found that using a micro-enema before rectal DWI can reduce artifacts and improve assessment of residual tumors. In another study, Lee et al. [28] mentioned an efficient [^18^F]FDG-PET/MRI protocol including Dixon-VIBE, T1-weighted, and T2-weighted images targeted to characterize liver tumors and lesions. Their proposed protocol takes about 30 min and they reported 95.7% sensitivity for all primary tumors. The PET/Dixon-VIBE/T1/T2 MRI protocol was effective in TNM staging, with 7 out of 14 patients with distant metastases detectable. While three-dimensional T2-weighted MRI can be useful for evaluating neoadjuvant therapy responses, 2D imaging is still preferred. T1-weighted imaging with a broader field of view may assist in evaluating distant lymph nodes and incidental pelvic findings, especially in mucinous neoplasms. Although contrast-enhanced T1-weighted imaging does not enhance local staging accuracy, it may help identify local recurrence during restaging with heterogeneous enhancement indicating recurrence [29]. Table 1 and Table 2 provide a summary of the key MRI parameters for the most used rectal MRI sequences across major MRI equipment vendors and some of the key findings from studies on MRI sequences used for rectal cancer imaging.

### 3.3. PET/MRI in Rectal Cancer Staging

The TNM classification for rectal cancer categorizes the tumor based on its extent (T), lymph node involvement (N), and distant metastasis (M). T1 tumors invade the submucosa, T2 invade the muscularis propria, T3 extend into perirectal tissues, and T4 tumors invade either the visceral peritoneum (T4a) or adjacent organs (T4b). Lymph node involvement is classified as N0 (no lymph node metastasis), N1 (1–3 regional nodes), and N2 (≥4 regional nodes). Distant metastasis is defined as M0 (none), M1a (single distant organ/site), M1b (multiple distant organs/sites), and M1c (peritoneal metastases). This TNM classification plays a significant role in prognosis assessment and treatment planning. A key anatomical landmark in rectal cancer staging is the distance to or involvement of the mesorectal fascia, which determines the feasibility of complete tumor resection. MRI is the gold standard for assessing tumor involvement with the mesorectal fascia, providing essential information for surgical and non-surgical treatment decisions [30]. MRI is less effective in detecting distant metastasis and lymph node characterization. This may be a benefit of advanced imaging techniques like [^18^F]FDG-PET/MRI for tumor staging. [^18^F]FDG-PET/MRI can improve nodal staging accuracy, distinguishing metastatic from reactive lymph nodes. [^18^F]FDG-PET/MRI improves the detection of distant metastases, particularly in the liver and peritoneum (M1b/M1c), which may be missed by MRI alone. The following sub-sections give an overview of the existing literature in primary, nodal, and metastasis staging.

#### 3.3.1. Primary Tumor Staging

MRI in preoperative staging identifies the tumor location, assesses the mesorectal fascia, and differentiates tumor invasion from fibrosis. This is important for guiding treatment decisions, as more locally advanced tumors may benefit from neoadjuvant chemoradiation therapy. MRI’s accuracy in T-staging remains limited (67–86%), particularly in differentiating T1/T2 from early T3 tumors and distinguishing true mesorectal invasion from desmoplastic reactions. A study by Gagliardi et al. [31] reported MRI’s sensitivity, specificity, and accuracy for detecting invasion through the rectal wall as 89%, 80%, and 86%, but showed limitations in distinguishing T1-T2 from early T3 tumors, while N staging showed moderate reliability, with 67% sensitivity, 71% specificity, and 69% accuracy. In another study related to T-staging of rectal tumors, Brown et al. [32] have also shown disagreements in staging between T1 and T2 tumors, and between T2 and T3 tumors, where MRI results were correlated with histopathological findings. Similar results were observed in the study by Poon et al. [33] which compared MRI with histopathological findings. MRI showed an overall diagnostic accuracy of 74%, with sensitivity and specificity of 62% and 79% for pT2, 84% and 59% for pT3, and 50% and 76% for pT4 lesions, and demonstrated a strong accuracy in predicting lateral resection margin (LRM, equivalent to circumferential resection margin) status. In the study by Blomqvist et al. [34], MRI of resected rectal specimens showed a sensitivity, specificity, positive predictive value, and negative predictive value of 88%, 78%, 64%, and 93%, respectively, for detecting tumor involvement when LRM was ≤1 mm. MRI measurements of LRM were shorter than histopathological findings in eleven cases but correctly identified seven of eight cases with non-radical excision. However, MRI was unreliable in predicting lymph node metastases. In a meta-analysis conducted by Hoeffel et al. [25] which excluded patients treated with preoperative radiotherapy or chemoradiotherapy, MRI was found to be quite accurate in assessing both the circumferential resection margin (CRM) and tumor stage (T category). MRI results showed sensitivities of 77% and 94% for CRM, and 87% and 75% for T category with a high specificity for CRM.

Integrating [^18^F]FDG-PET with MRI should enhance lesion detection and improve margin delineation of the primary tumor. In their review, Rosenkrantz et al. [35] highlighted that simultaneous [^18^F]FDG-PET/MRI offers better characterization of tumor extension beyond the muscularis propria. Among the few studies addressing this, Catalano et al. [36] showed that while MRI alone correctly identified T stage in 56 out of 62 cases, [^18^F]FDG-PET/MRI improved accuracy, correctly staging 60 out of 62 cases—misinterpreting only 1 T2 case as T3 and 1 T4 case as T3. However, there is increased focus on impact of restaging after preoperative chemoradiation with [^18^F]FDG-PET or CT or MRI scan. In their study, Schneider et al. [37] discussed the challenges of restaging where the accuracy of restaging in all metastatic disease was <80%. They showed the impact of each restaging modality, with PET—11%, CT—4%, and MRI—4%. In metastatic disease at primary staging, the impact of restaging was reported as PET—32%, CT—18% and MRI—6%. This suggests that changes in the extent of disease after chemoradiation result in changes in overall disease management, with PET showing a significant impact in restaging. Table 3 below highlights some studies and their key findings with regard to rectal cancer staging of the primary tumor.

#### 3.3.2. Nodal Staging

**Figure 2:**
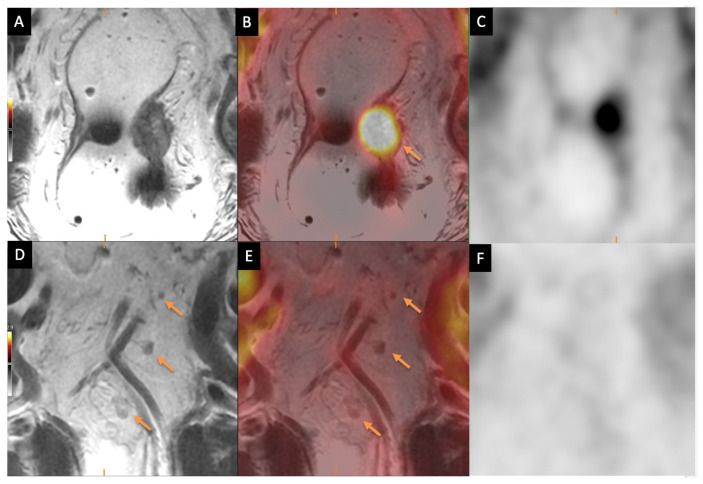
Rectal cancer staging with PET/MRI. (**A**) Initial MRI shows a T3 rectal adenocarcinoma centered at the high left rectal wall. (**B**) Fused [^18^F]FDG-PET/MRI images show focal FDG activity (arrow). (**C**) PET only images confirm focal activity in the rectal tumor. (**D**) MRI shows small nonspecific lymph nodes (arrows). (**E**) Fused [^18^F]FDG-PET/MRI images show no focal activity in lymph nodes, suggesting likely reactive and non-malignant nodes (arrows). (**F**) Corresponding PET-only images confirm no focal activity in lymph nodes.

Accurate lymph node assessment remains a significant challenge in rectal cancer MRI, particularly in preoperative staging. While lymph nodes larger than 1 cm are more reliably identified as malignant, the specificity of MRI decreases for smaller nodes, especially those under 5 mm, leading to potential understaging. This limitation is particularly relevant in patients undergoing neoadjuvant therapy, as shown in a study by Langman et al. [43], which found that not only does neoadjuvant treatment reduce the total lymph node yield in resection specimens, but it also alters the distribution of nodes, further complicating assessment and staging. Figure 2 demonstrates how [^18^F]FDG-PET/MRI can help improve specificity of otherwise small, nonspecific lymph nodes.

A study led by Lahaye et al. [44] highlighted the diagnostic performance of MR imaging considering nodal restaging in 39 patients treated with radiation therapy, restaging and resection. A total of 320 mesorectal nodes were found in histopathological evaluation after radiation therapy, and 325 nodes after surgical resection, out of which 201 matched with MR findings. The specificity of malignant lymph nodes was reported as 85% and 78% for reader 1 and reader 2. A study by Kim et al. [45] highlighted a comparison between MRI and [^18^F]FDG-PET/CT for preoperative nodal staging in rectal cancer patients. Out of 30 patients, metastatic mesorectal lymph nodes were found in 18 patients and MRI showed 83% accuracy, 94% sensitivity, and 67% specificity, whereas [^18^F]FDG-PET/CT showed 70% accuracy, 61% sensitivity, and 83% specificity and combined MRI and [^18^F]FDG-PET/CT showed 90% accuracy, 94% sensitivity, and 83% specificity. Integrating PET with MRI may enhance the characterization of small pelvic lymph nodes. Several studies highlight the superior diagnostic value of [^18^F]FDG-PET for nodal assessment. Cerny et al. [46] compared [^18^F]FDG-PET/CT (SUV_max_, SUV_mean_) with DWI-MRI (ADC_min_, ADC_mean_) in rectal cancer staging. Their analysis of 44 pathological and 19 control lymph nodes found no significant size difference. However, pathological nodes exhibited higher SUV_max_ and SUV_mean_ on PET/CT and lower ADC_mean_ on MRI, differentiating them from control nodes (*p* < 0.01). ADC_min_ did not show a significant difference, suggesting its limited role in nodal characterization. A similar study was also reported by Jeong et al. [47] which compared the correlation between ADC and SUV uptake values derived from hybrid [^18^F]FDG-PET/MRI and [^18^F]FDG-PET/CT. They reported inverse correlation between [^18^F]FDG-PET and water diffusion on DWI with lower SUV than [^18^F]FDG-PET/CT. This decrease may be due to a difference in the attenuation correction methods in [^18^F]FDG-PET/MRI and [^18^F]FDG-PET/CT. DWI MRI (b = 1000) for nodal characterization has high sensitivity but moderate specificity, and reported 93% sensitivity and 81% specificity. For example, in one study of 1030 benign lymph nodes, 197 were reported as false positives [48]. Extended PET acquisition times in PET/MRI improve the detection rates of metastatic lymph nodes in rectal cancer patients [49]. A total of 94 abnormal lymph nodes were identified on PET, all with corresponding MRI anatomic correlates. Among these, 37 nodes (39.4%) were detected exclusively during the 15 min dedicated acquisition. Additionally, 57 nodes (60.6%) were 5 mm or smaller, with 29 (30.9%) only visible on the 15 min acquisition. A total of 31 nodes (33.0%) measured between 5.1 and 10 mm, with 8 (25.8%) detected only during the 15 min acquisition. Among the 17 subjects imaged for initial staging, 11 (64.7%) were upstaged due to the extended PET acquisition time, including 10 cases from N1 to N2 and 1 from N0 to N1. This demonstrates that extending the PET scan times during [^18^F]FDG-PET/MRI not only helps detect more lymph nodes but also enhances the overall effectiveness of [^18^F]FDG-PET/MRI in detecting and characterizing lesions. Ince et al. [14] have highlighted the results based on [^18^F]FDG-PET/MRI performed for initial staging, post-TNT staging, and surveillance during the non-operative management stage. The study reported [^18^F]FDG-PET/MRI showing 100% accuracy for clinical complete response (cCR) at post TNT as compared to MRI alone. [^18^F]FDG-PET/MRI was found to have improved locoregional staging of rectal cancer [41]. In this study comprising 46 patients, MRI correctly identified T stage in 27/46 and N stage in 32/46 patients correctly. MRI alone correctly identified 32/46 LARC cases. Combined [^18^F]FDG-PET/MRI reported a sensitivity of 90% for T-stage, 63% for N-stage, and 95% for LARC, and a specificity of 50% for T-stage, 76% for N-stage, and 50% for LARC. Table 4 describes the overviews of various studies and their key findings in nodal staging of rectal cancer.

#### 3.3.3. Distant Metastasis Staging

MRI is considered the gold standard for hepatic metastasis assessment, particularly with the ability to utilize hepatobiliary contrast agents [55]. [^18^F]FDG-PET/MRI enhances hepatic staging by combining MRI’s superior soft tissue contrast with PET’s metabolic imaging, which improves lesion detection and characterization. Hepatobiliary phase (HBP) MRI is the preferred modality for detecting liver metastases, particularly those arising from colorectal cancer [56]. Studies have shown that whole-body [^18^F]FDG-PET/MRI provides added value over contrast-enhanced CT for identifying and characterizing metastatic lesions [57]. A study by Park et al. [56] compared diffusion-weighted imaging (DWI) combined with T2-weighted imaging and T2-weighted imaging alone for assessing tumor invasion in the mesorectal fascia (MRF) in rectal cancer. The diagnostic accuracy of combined DWI and T2-weighted imaging is reported to be 89%, sensitivity 94% and specificity 97% whereas with T2-weighted imaging alone is 40% accuracy, sensitivity 29% and specificity 30%. This highlights the significant value of integrating DWI with T2-weighted imaging for more accurate assessment of tumor spread in rectal cancer. Goh et al. [58] discussed DCE-MRI of the liver, using high-temporal-resolution T1-weighted imaging to track contrast bolus passage and enable quantification of hepatic perfusion. Their review on liver MRI also recommends free-breathing, multiple-averaging DW-MRI with fat-suppressed spin-echo echo-planar techniques as the most widely used approach. In another study, Seto et al. [59] highlighted a [^18^F]FDG-PET/MRI protocol without usage of contrast where they used early delayed and extended dedicated pelvic MRI for a period of 80 min and 15 min after FDG injection. The accuracy for liver and lung metastasis was reported to be 100% (four out of four patients with liver metastasis were detected). They reported high sensitivity for [^18^F]FDG-PET for tumor glycolytic activity. There are also studies which suggest the superiority of PET/MRI for detecting liver metastasis, where [^18^F]FDG-PET/MRI with DWI showed 99% and contrast-enhanced MRI showed 98%, and both showed 99% accuracy [60]. [^18^F]FDG-PET/MRI with contrast was found to be more specific for detecting extrahepatic malignancies, assessment of perirectal and mesorectal disease, and in evaluation of distant metastases [61,62,63]. Table 5 highlights the existing studies on metastasis staging.

### 3.4. PET/MRI for Response Assessment in Rectal Cancer

In recent years, mortality rates related to rectal or colorectal cancer have declined due to significant advancements in therapeutic management, including the implementation of both adjuvant and neoadjuvant therapies [69]. Neoadjuvant therapy (NAT) enables sphincter-preserving surgery for patients who show a strong response to treatment. Tumor response to NAT is a key prognostic factor in LARC. While 10–40% of patients achieve a clinical complete response (cCR) after NAT, true pathological complete response (pCR) rates are approximately half as frequent [70]. Restaging requires comparing post-treatment images with pre-treatment images, maintaining consistency in the imaging protocol and planes used for both [71]. This decrease in accuracy can be attributed to challenges such as overstaging of nodal disease, difficulty in distinguishing between tumoral infiltration or residual tumor and desmoplastic reaction or radiation fibrosis, and the potential misinterpretation of radiation proctitis as local invasion [72]. Ruggieri et al. [73] mentioned, with regard to evaluating mucinous adenocarcinomas on post-treatment MRI, that it happens to be difficult since these tumors remain hyperintense on T2-weighted images, regardless of treatment response. Restaging also relies on reassessing the mesorectal fascia (MRF), which plays a key role in evaluating treatment response. A study by Van Der et al. [74] highlighted MRI that demonstrated a sensitivity of about 76% and a specificity of 86% in assessing the MRF within the irradiated pelvis. The accuracy of MRI for restaging tends to be lower compared to its initial staging accuracy.

Nonetheless, MRI has emerged as a valuable tool in assessing treatment response in rectal cancer, particularly through MRI-based tumor regression grading (mrTRG). Patel et al. [24] highlighted in the MERCURY trial that mrTRG serves as a significant predictor of survival outcomes. Their findings demonstrated that poor mrTRG was independently associated with worse overall survival and disease-free survival. Patients with poor mrTRG had a five-year overall survival rate of 27% compared to 72% for good responders (*p* = 0.001), while their disease-free survival was also significantly lower (31% for poor vs. 64% for good responders, *p* = 0.007). As MRI T-staging and tumor regression grade (mTRG) are useful tools for predicting how well a patient responds to treatment and the likelihood of tumor recurrence. The study also found that among the 111 patients, those with CRM involvement were more likely to have poor treatment response (classified as ypT-poor). Of the 23 patients with a positive CRM, 21 (or 91%) had tumors classified as poor responders, which indicates a higher risk of recurrence.

**Figure 3:**
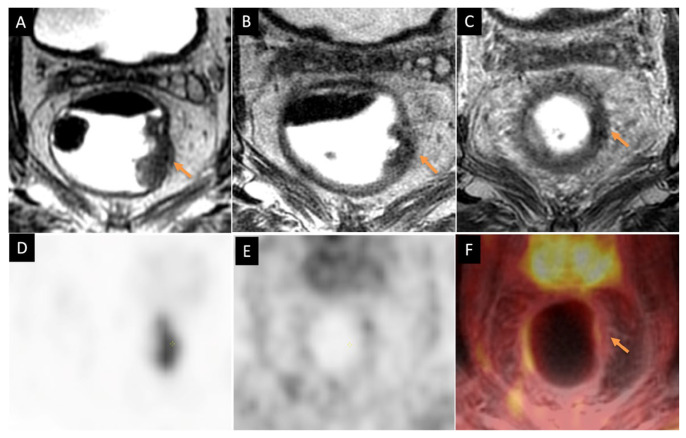
Rectal cancer treatment response assessment with PET/MRI. (**A**) Initial MRI showed a T3N1a rectal adenocarcinoma centered at the left mid rectal wall (arrow). (**B**) MRI after treatment with radiation therapy 25Gy in 5 fractions shows residual tumor with slight response (arrow). (**C**) MRI from PET/MRI exam after completion of total neoadjuvant therapy (8 cycles of FOLFOX) shows possible complete response and heterogeneous scar in the left wall (arrow). (**D**) Corresponding dedicated pelvic PET only image acquired for 15 min shows focal activity prior to treatment. (**E**) Post treatment dedicated PET only image acquired for 15 min shows no focal activity at the previous tumor site. (**F**) Fused PET/MRI (15 min PET bed fused to post contrast T1-weighted MRI) indicated complete response at the previous tumor site (arrow).

There are studies reporting better accuracy with [^18^F]FDG-PET/MRI than MRI alone [75]. Crimì et al. [11] reported PET/MRI accuracy higher than MRI for both ypT and ypN staging. Figure 3 shows an example of the improved specificity achievable with [^18^F]FDG-PET/MRI compared to MRI alone in assessment of complete response. [^18^F]FDG-PET/MRI, due to combined metabolic and anatomic information, can improve the tumor detection rate, reducing the risk of staging or understaging compared to MRI alone. Cerny et al. [76] have mentioned in their study about the role of [^18^F]FDG-PET/MRI in treatment response assessment of rectal cancer patients who are undergoing total neoadjuvant therapy. The [^18^F]FDG-PET provided more diagnostic accuracy than MRI alone. This was found in 80% of cases. While [^18^F]FDG-PET/MRI is advantageous for soft tissue contrast and local tumor assessment, a study led by Rutegård et al. [62] demonstrated that [^18^F]FDG-PET/CT led to upstaging, detection, and treatment in a patient with a liver metastasis before rectal cancer surgery, while another patient had a small active lung nodule, although it did not alter their treatment plan. A study by Capirci et al. [77] shows that PET scans can help predict how well rectal cancer patients respond to chemotherapy and radiation before surgery. The study found that patients who responded well had a decrease in SUV_max_ after treatment, which was a good indicator of treatment success. [^18^F]FDG-PET/MRI, however, did not impact patient management in any of these cases. Table 6 gives an overview of studies related to [^18^F]FDG-PET/MRI response assessment for rectal cancer imaging. Figure 3 demonstrates a rectal cancer case of response assessment with PET/MRI.

### 3.5. PET Tracers in Rectal Cancer

While [^18^F] fluorodeoxyglucose or [^18^F]FDG is the most widely used PET tracer, novel tracers targeting tumor hypoxia, proliferation, fibrosis, and receptor expression also rely on SUV to quantify uptake patterns. SUV in [^18^F]FDG-PET/CT correlates with tumor response, where a significant SUV reduction after treatment predicts favorable outcomes. [^18^F]FDG-PET is also used for assessing tumor response to therapy, particularly with molecular inhibitors. Traditional RECIST criteria, based on tumor size, have limitations, especially with non-cytotoxic treatments like tyrosine kinase inhibitors [91]. In gastrointestinal stromal tumors, [^18^F]FDG-PET has demonstrated early metabolic response to imatinib correlating with patient outcomes [92]. [^18^F]FDG-PET has shown promise in predicting treatment response in metastatic colorectal cancer, with a 70% correlation between metabolic changes and liver metastasis necrosis following irinotecan and bevacizumab therapy [93]. Similar findings have been observed in rectal cancer patients undergoing neoadjuvant chemoradiotherapy. Early data suggest that lapatinib, an Erb-B1 and Erb-B2 inhibitor, reduces metabolic activity in responders that help in predicting tumor response.

However, there is limited clinical evidence on FDG-PET/CT for monitoring EGFr inhibitors and tumor regression in patients with rectal cancer after neoadjuvant chemoradiotherapy has been reported. In a study led by Arulamplam et al. [94], they compared a new PET tracer, [^18^F]3′-deoxy-3′-fluorothymidine (^18^FLT), and FDG in patients with colorectal cancer. They found that ^18^FLT shows a high sensitivity (98%) in the detection of extrahepatic disease but poor sensitivity (34%) for the imaging of colorectal liver metastases compared to ^18^FDG. Another study by Puri et al. [95] has demonstrated the use of [^18^F]fluoromisonidazole [^18^F]FMISO PET tracer for rectal cancer patients receiving neoadjuvant CRT. Hypoxia in cancer cells contributes to radioresistance, which can affect radiotherapy (RT) outcomes, so current radiation strategies aim to target hypoxia. Tumor hypoxia arises from multiple factors, including impaired oxygen supply related to perfusion, diffusion, or anemia. [^18^F] FMISO PET has shown promising results in detecting tumor hypoxia correlating with pO2 levels in head and neck cancers. There are still studies that need to be performed exploring [^18^F] FMISO PET changes before and after neoadjuvant chemoradiotherapy (CRT) in rectal cancer to assess treatment response. Wieder et al. [96] examined the use of PET imaging with [^18^F]3′-deoxy-3′-fluorothymidine (FLT) to track the effectiveness of preoperative chemoradiotherapy in rectal cancer. Before therapy, tumor FLT uptake was 4.2 SUV; after 14 days of chemoradiotherapy, FLT uptake dropped to 2.9 SUV (28.6% reduction); and before surgery (after completing neoadjuvant therapy), FLT uptake further decreased to 1.9 SUV (54.7% reduction). The researchers found that although the FLT uptake in tumors decreased, it did not correlate with histopathological findings for tumor regression, and hence, FLT-PET did not seem to be a promising tracer for rectal tumor assessment. Table 7 highlights the key findings associated with PET tracers and rectal cancer.

## 4. Discussion

PET/MRI is emerging as a powerful imaging modality in rectal cancer staging, response assessment, and guiding treatment decisions. PET/MRI represents a significant advancement in rectal cancer imaging by enhancing locoregional staging through precise assessment of mesorectal fascia involvement, lymph node status, and extramural vascular invasion (EMVI). Compared to PET/CT, PET/MRI provides superior soft tissue contrast, enabling a more accurate evaluation of tumor extent. This improved imaging capability is particularly beneficial in preoperative planning, as it aids in determining whether a patient is a candidate for total mesorectal excision (TME) or requires neoadjuvant therapy for tumor downstaging. Additionally, PET/MRI plays a crucial role in distinguishing early responders from non-responders, allowing for timely treatment modifications, and potentially avoiding unnecessary surgery in cases of complete response. Despite these benefits, PET/MRI is associated with several technical and clinical challenges. Longer scan durations may lead to patient discomfort and increase the risk of motion artifacts. Moreover, attenuation correction remains a limitation, potentially affecting standardized uptake value (SUV) quantification and the reproducibility of metabolic measurements.

A major challenge remains in distinguishing reactive lymph nodes from metastatic ones, which affects the accuracy of nodal staging and the staging of liver metastasis and distal metastatic disease. PET/MRI potentially can address all these needs. For mesorectal staging, combining diffusion-weighted imaging (DWI) with T2-weighted MRI significantly improves accuracy, with studies reporting 89% diagnostic accuracy, compared to 40% with T2-weighted imaging alone. This highlights the need for incorporating DWI into routine rectal cancer staging to enhance preoperative planning. However, this review highlights the limited research on response assessment using PET/MRI, underscoring the need for further investigation.

PET/MRI has shown promise in distinguishing post-treatment fibrosis from residual tumors, which is crucial for evaluating clinical complete response (cCR) after neoadjuvant therapy (NAT). PET/MRI enhances restaging accuracy data and reducing the risk of over- or understaging. Studies highlight PET/MRI’s potential to refine the “Watch and Wait” strategy, minimize overtreatment, and improve patient selection for non-surgical management. However, the high cost and limited availability of PET/MRI continue to restrict its widespread adoption in clinical practice. Another challenge related to PET/MRI is FDG, which acts as the primary PET tracer. Although it is widely available, it lacks specificity in post-treatment assessment due to inflammation-related uptake. This limitation has led to growing interest in alternative tracers such as FLT for assessing tumor proliferation and FAPI (fibroblast activation protein inhibitor) for detecting tumor-associated fibroblasts. These novel tracers have the potential to improve the accuracy of response assessment and in differentiating residual tumor from post-treatment fibrosis. However, further clinical validation is required before they can be integrated into routine practice. Therefore, future research should focus on standardizing PET/MRI protocols to improve their clinical utility. The integration of artificial intelligence and radiomics in PET/MRI analysis holds promise for enhancing lesion characterization, predicting therapy responses with greater accuracy, shortening scan times, and reducing costs.

Most available studies on PET/MRI in rectal cancer are retrospective and involve relatively small heterogenous patient populations. There lies variability in imaging protocols, acquisition parameters, interpretation criteria, etc., which limits comparability across studies. These factors reduce the generalizability of the current evidence and highlights the need for multicenter prospective trials with standardized protocols. Hence, future research should focus on standardizing PET/MRI protocols to improve their clinical utility. This effort will confirm the clinical value of PET/MRI in treatment establishment for rectal cancer.

## 5. Conclusions

There is literature highlighting the superiority of PET/MRI over conventional imaging for staging, restaging, treatment response evaluation, and follow-up in colorectal cancers. In clinical settings, the role of PET/MRI in rectal cancer is still being established, but it shows promise in both initial staging and treatment response assessment. Integration of hepatobiliary imaging and diffusion-weighted imaging can enhance PET/MRI’s diagnostic value. Factors such as scanner availability and integration into clinical trials could influence the future role of PET/MRI in rectal cancer staging. In summary, with the ongoing advancements in imaging hardware and software and the introduction of novel PET tracers, PET/MRI is expected to play an increasingly vital role in the comprehensive management of rectal cancer patients.

## Figures and Tables

**Table 1:** Commonly used rectal MRI parameters. Here, field of view is measured in millimeters and TR/TE is measured in milliseconds; TE = echo time, TR = repetition time, and TSE = turbo spin-echo.

Sequence	Vendor	Field Strength	TR (ms)	TE (ms)	Other Parameters
T2W FSE (Axial, Sagittal, Coronal)	GE	1.5/3 T	4000–6000	100–120	PROPELLER to reduce motion artifacts
	Siemens	1.5/3 T	3000–5000	80–110	Turbo spin-echo (TSE), no fat suppression
	Philips	1.5/3 T	4000–6000	90–130	TSE, high-resolution imaging
	United Imaging	1.5/3 T	4000–6000	90–120	TSE, motion correction (uCS compressed sensing optional)
T2W FSE (Large FOV Axial)	GE	1.5/3 T	5000–8000	90–130	Full pelvis coverage
	Siemens	1.5/3 T	4000–7000	90–120	TSE, large field of view
	Philips	1.5/3 T	5000–8000	100–140	TSE, no fat suppression
	United Imaging	1.5/3 T	4500–7500	90–130	TSE, large FOV, optional fat sat
DWI (b ≥ 800 sec/mm^2^)	GE	1.5/3 T	3000–5000	Min Full	EPI-based, Fat suppression
	Siemens	1.5/3 T	3000–4500	Min Full	RESOLVE for motion correction
	Philips	1.5/3 T	3000–5000	Min Full	DIXON for fat suppression
	United Imaging	1.5/3T	3000–5000	Min Full	EPI-based DWI, DWI with distortion correction, fat suppression
T1W Imaging (Wide FOV)	GE	1.5/3 T	500–800	10–15	Covers distant nodes, incidental findings
	Siemens	1.5/3 T	500–900	10–20	TSE for soft tissue contrast
	Philips	1.5/3 T	500–900	10–18	dS TSE for optimized signal
	United Imaging	1.5/3 T	500–850	10–18	TSE, uCS accelerated, wide coverage

**Table 2:** Summary of key findings from studies on MRI sequences in rectal cancer imaging.

Author	Year	Study Design	Sample Size	Study Objective	Key Findings Related to MRI Sequences
Beets et al. [23]	2018	Expert-based consensus study	14 abdominal imaging experts participated in a consensus meeting where 246 questionnaire items were scored and classified as ‘appropriate’ or ‘inappropriate’.	Demonstrates the 2012 ESGAR consensus guidelines on the MRI acquisition and reporting for clinical staging and restaging of rectal cancer.	85% of the panel agrees for use of DWI in T-stage assessment; 54% agrees for DWI alone for complete response detection; they agreed on no current role of ADC quantification due to lack of standardization; the panel reached full consensus for qualitative assessment of DWI and ADC maps; DCE-MRI should be considered a research tool, not for routine use
Hoeffel et al. [25]	2014	Systematic review based upon continuing education program	Meta-analysis from 2000–2011	Focuses on assessment of the initial locoregional extension of rectal tumors. It also highlights spasmolytic agents in case of tumor delineation.	Spasmolytic agents allow better tumor delineation and reduce artifacts in diffusion-weighted acquisition. Rectal tumors are accurate in T2-weighted MR. T2-weighted sequences without fat suppression in the three planes and perpendicular to the axis of the tumor are essential and sufficient
Messina et al. [26]	2020	Systematic review	An up-to-date review	Focuses on tissue signal attenuation with increasing b values.	DWI with b values 1000 can be helpful in primary staging of rectal cancer in adjunction to conventional sequences
Van Griethuysen et al. [27]	2018	Retrospective study	50 rectal cancer patients from 2012 to 2016 who underwent DWI MRI	Focuses on protocol usage of micro-enema in EPI-DWI and its effects related to susceptibility artifacts in DW imaging of the rectum at 1.5 T.	First study to show that application of a micro-enema shortly before image acquisition reduces susceptibility artifacts on rectal DWI
Lee et al. [28]	2015	Retrospective study	59 colorectal patients	To investigate whole-body FDG PET/Dixon-VIBE, T1-weighted, and T2-weighted MRI protocols	PET/Dixon-VIBE/T1/T2 MRI protocol is clinically useful for TNM staging (95.7% sensitivity) and chemonaive hepatic metastasis
Sinaei et al. [29]	2013	Retrospective study	42 patients with pelvic recurrence of rectal cancer from 1998 to 2009	Focuses on identification of pelvic recurrence and evaluation of T2 signal intensity to differentiate tumor with fibrosis.	Recurrence commonly occurs in the anterior pelvic structures, sidewall, presacral space, and pelvic floor. An intermediate or high T2 signal and soft-tissue enhancement beyond six months post-surgery should raise suspicion for recurrence

**Table 3:** Summary of key findings from studies on primary tumor staging in rectal cancer patients.

Author	Year	Study Design	Sample Size	Study Objective	Key Findings
Crimì et al. [11]	2021	Retrospective study	36 patients with LARC	To assess FDG-PET/MRI for TNM restaging	[^18^F]FDG-PET/MRI reported a sensitivity of 94–100%, specificity of 73–94%, and accuracy of 92–100% for T-staging; for N staging, sensitivity, specificity, and accuracy were 90–93%, 92–94%, and 42–90%; for M staging, [^18^F]FDG-PET/MRI showed 96% sensitivity, 80% specificity, and 74% accuracy
Patel et al. [24]	2011	Prospective study	111 rectal cancer patients treated by neoadjuvant therapy between February 2002 and October 2003	To determine and correlate MRI assessment of TRG and CRM with pathological staging	Both MRI T-staging and tumor regression grade (mTRG) showed a statistical correlation with ypT (post-treatment histopathological T stage). Among 23 of 111 patients with a positive circumferential resection margin (CRM), 21 (91%) had tumors classified as ypT-poor, indicating poor treatment response and higher recurrence risk
Gagliardi et al. [31]	2002	Retrospective study	28 rectal cancer patients who did not undergo irradiation	Focuses on comparing MRI with pathological staging	MRI for bowel wall invasion: Sensitivity 89%, specificity 80%, and accuracy 86%, indicating high reliability in detecting tumor extension beyond the bowel wall. MRI for Malignant Lymphadenopathy: sensitivity 67%, specificity 71%, and accuracy 69%, showing moderate performance in identifying metastatic lymph nodes
Brown et al. [32]	2003	Retrospective study	98 patients undergoing total mesorectal excision	To determine accuracy of preoperative MRI in evaluating pathological and surgical prognostic factors related to local recurrence	Study highlights clinical case where T2-weighted fast spin-echo can identify extramural vascular expansion which is confirmed in histological WSI. The same has been found for prediction of circumferential resection margin and tumor perforation through peritoneum. MRI showed cases where T1-T2 tumors were staged as T2 and eight pT2 tumors were staged as T3
Poon et al. [33]	2005	Retrospective study	42 rectal cancer patients who underwent surgical resection for primary tumor	To evaluate the accuracy of T2-weighted MRI.	MRI correctly predicted 31 out of 42 histopathological T-stage lesions with an accuracy of 74%. MR sensitivity and specificity was 62% and 79% for pT2 lesions, 84% and 59% for pT3 lesions. and 50% and 76% for pT4 lesions
Blomquist et al. [34]	1997	Retrospective study	26 patients who underwent MRI after mesorectal excision	To investigate whether MRI can predict tumor involvement of lateral resection margin	Presence of tumor-free lateral resection margin can be predicted by MRI when it exceeds 1 mm
Catalano et al. [36]	2021	Retrospective study	62 rectal cancer patients	To assess [^18^F]FDG-PET/CT with CT and MRI alone in clinical staging	For local staging, sensitivity, specificity, and accuracy were reported as 79%, 78%, and 73% for CT; 86%, 77%, and 82% for MRI; and 90%, 76%, and 85% for [^18^F]FDG-PET/CT; for distant staging, sensitivity, specificity, and accuracy were reported as 89%, 94.9%, and 77% for CT; 85%, 99%, and 98% for MRI; and 86%, 97.2%, and 83% for [^18^F]FDG-PET/CT
Schneider et al. [37]	2016	Retrospective study	199 rectal cancer patients	To assess changes in staging observed with PET-CT, CT, and MRI before and after neoadjuvant chemoradiotherapy	T stage was accurate on restaging MRI in 43% patients, with 34% patients overstaged and 18% understaged. In N stage, accuracy of MRI and PET was 56% and 60%
Bamba et al. [38]	2011	Retrospective study	256 CRC including preoperative and postoperative patients	To assess sensitivity and specificity of FDG-PET/CT for diagnosing local recurrence compared to CT/MRI	95.5% patients with local recurrence had positive [^18^F]FDG uptake by PET/CT with 4.5% negative results; CT/MRI suspected 45.5% cases to be positive and 4.5% negative. PET/CT had 95.5% sensitivity and 100% specificity
Li et al. [39]	2020	Retrospective study	34 patients with rectal cancer	To investigate diagnostic performance of [^18^F]FDG-PET/MRI and MRI alone for staging and restaging of rectal cancer patients	Both [^18^F]FDG-PET/MRI and MRI alone showed similar accuracies for locoregional T-staging; for N staging, combined [^18^F]FDG-PET/MRI showed a sensitivity of 93.8% and specificity of 91.7% compared to MRI alone (sensitivity 93.8%, specificity 83.3%); in distant metastases, combined PET/MRI had lower sensitivity (82.6%) and specificity (87%) than MRI alone (sensitivity 87%, specificity 91.3%)
Hotta et al. [40]	2018	Retrospective study	59 rectal cancer patients	To assess diagnostic performance of [^18^F]FDG-PET/CT using point spread function reconstruction over conventional PET/CT on initial staging	For N staging, PSF-PET/CT showed higher sensitivity (78.6%) than conventional PET/CT (64.3%) and MRI (57.1%). Accuracy of T-staging was 69.4% for PSF-PET/CT compared to conventional PET/CT (73.5%) and MRI (73.5%). In M staging, both PSF-PET/CT and conventional PET/CT diagnosed all distant metastases accurately
Herold et al. [41]	2022	Retrospective study	46 patients with primary rectal cancer	To determine whether combined PET/MRI can improve LARC and to assess its prognostic value after resection	For T-staging, PET/MRI showed a sensitivity and specificity of 90% and 50% compared to MRI sensitivity (55%) and specificity (65.4%). For N staging, PET/MRI showed a sensitivity and specificity of 63.6% and 76.5% compared to MRI sensitivity (72.7%) and specificity (70.6%)
Cho et al. [42]	2009	Retrospective study	30 rectal cancer patients	To investigate accuracy of MRI and [^18^F]FDG- PET/CT for restaging after preoperative CCRT	Overall accuracy of MRI for T and N staging was 67% and 75%; [^18^F]FDG-PET/CT was 60% and 71%; [^18^F]FDG-PET/CT identified distant metastases with 97% accuracy

**Table 4:** Summary of key findings from studies on nodal staging in rectal cancer patients.

Author	Year	Study Design	Sample Size	Study Objective	Key Findings
Ince et al. [14]	2022	Retrospective study	14 rectal cancer patients	To assess [^18^F]FDG-PET/MRI at initial and post-TNT restaging	[^18^F]FDG-PET/MRI after post TNT can detect more residual diseases than MRI alone and provided added value to 82% restaging cases
Lahaye et al. [44]	2009	Retrospective study	39 patients with rectal cancer	To determine diagnostic performance of nodal restaging using MRI after radiation therapy	2D T2-weighted fast spin-echo images could be accurately matched with 201 lymph nodes (40 malignant; 285 benign) out of 325 lymph nodes
Kim et al. [45]	2011	Retrospective study	30 rectal cancer patients who underwent surgery from September 2008 to March 2009	To compare MRI with [^18^F]FDG-PET/CT for preoperative assessment of nodal staging	MRI predicted nodal status with 83% accuracy, 94% sensitivity and 67% specificity. [^18^F]FDG-PET/CT had 70% accuracy, 61% sensitivity, and 83% specificity. Combining MRI and [^18^F]FDG-PET/CT improved overall accuracy to 90%, with 94% sensitivity and 83% specificity
Cerny et al. [46]	2016	Retrospective study	27 patients with locally advanced rectal cancer from October 2012 to September 2014	To compare DWI-MRI with [^18^F]FDG-PET/CT in LARC patients	Pathological nodes exhibited higher SUV_max_ and SUV_mean_ on FDG-PET/CT and lower ADC_mean_ on MRI
Jeong et al. [47]	2016	Retrospective study	9 patients with confirmed rectal adenocarcinoma	To assess correlation between SUV and ADC values associated with [^18^F]FDG-PET/MRI	There is an inverse correlation between SUV of [^18^F]FDG-PET/MRI and ADC of DWI
Heijnen et al. [48]	2013	Retrospective study	21 rectal cancer patients underwent surgery	To compare DWI MRI in lymph node characterization	DWI with b1000 detected 129/212 nodes compared to the 117/212 of T2-weighted MRI but could not identify metastatic nodes
Bailey et al. [49]	2018	Retrospective study	22 rectal cancer patients	To assess if extended PET acquisition in pelvis during [^18^F]FDG-PET/MRI improves metastasis lymph node staging	Out of a total 94 lymph nodes, extended 15 min PET acquisition detected 39.4% more abnormal lymph nodes (≤5 mm); 64.7% were upstaged on increased PET acquisition time
Chen et al. [50]	2023	Retrospective study	357 patients with 30.3% stage 3 rectal cancer and 71.7% receiving neoadjuvant therapy	To assess and compare PET findings with CT and MRI for accurate treatment management	PET/MRI helped provide a treatment strategy compared to CT alone or PET/CT in 21.6% patients; both PET/MRI and PET/CT showed overall accuracy in detecting peritoneal and lymph node metastases
Kam et al. [51]	2010	Retrospective study	23 patients with rectal adenocarcinoma	To compare [^18^F]FDG-PET with MRI in rectal cancer primary staging	MRI-PET fusion showed 44% sensitivity and 100% specificity for nodal assessment compared to FDG-PET (21% and 56%) and CT (25% and 60%)
Amori et al. [52]	2019	Retrospective study	42 rectal cancer patients	To assess staging performance of [^18^F]FDG-PET/MRI versus final oncologic stage	Compared with final oncologic stage, [^18^F]FDG-PET/MRI was correct in detecting 6/42(14%) liver lesions; in 2 patients who were candidates for peritonectomy, already in stage IV, PET/MRI disclosed multiple lesions in other organs, leading to abortion of operation
López et al. [53]	2024	Retrospective study	73 LARC patients	To compare MRI and PET/CT in nodal staging	MRI reported a sensitivity and specificity of 80% and 75% compared to PET/CT, with a sensitivity and specificity of 60% and 100%; PET/CT identified pelvic metastatic adenopathies in 8 patients not visible by MRI
Paspulati et al. [54]	2015	Retrospective study	12 rectal cancer patients	To compare hybrid [^18^F]FDG PET/MRI with PET/CT in staging and restaging	In 2 patients with rectal staging, both modalities showed no evidence of locoregional lymph node or distant metastasis. PET/CT showed the least accuracy in N and M staging and restaging—71% over 86% for PET/MRI; PET/CT failed to identify 3 lesions compared to PET/MRI

**Table 5:** Summary of key findings from studies on metastasis staging in rectal cancer patients.

Author	Year	Study Design	Sample Size	Study Objective	Key Findings
Seto et al. [59]	2022	Retrospective study	23 patients who underwent preoperative treatment	To assess [^18^F]FDG- PET/MRI without contrast for predicting metastasis	[^18^F]FDG-PET detected EMVI with irregular vessel and liver and lung metastasis with 100% accuracy
Queiroz et al. [60]	2020	Retrospective study	101 rectal cancer patients from November 2016 to April 2018	To assess diagnostic accuracy of [^18^F]FDG-PET/MRI for distant metastasis	[^18^F]FDG-PET/MRI reported 89.2% accuracy for liver and 76.4% accuracy for lung
Yoon et al. [61]	2019	Retrospective study	71 patients from January 2016 to August 2017	To compare contrast agent [^18^F]FDG-PET/MRI with contrast-enhanced CT	[^18^F]FDG-PET/MRI showed 98% specificity for metastasis staging
Rutegård et al. [62]	2019	Retrospective study	24 patients with 2 diagnosed with hepatic metastasis	To assess [^18^F]FDG-PET/CT for initial and restaging	The two patients were upstaged to M1 disease from [^18^F]FDG-PET/CT staging
Rutegård et al. [63]	2020	Retrospective study	27 patients	Assess [^18^F]FDG-PET/MRI results with histopathological findings	[^18^F]FDG-PET/MRI facilitates mesorectal structures and matches with histopathological findings than MRI alone
Akkus et al. [64]	2023	Prospective study	78 patients with CRC and liver metastasis	To evaluate diagnostic performance of [^18^F]FDG-PET/CT, PET/MRI and MRI alone	Sensitivity of 55.6%, 97.2%, and 100%; specificity of 98.5%, 100%, and 80.5%; and accuracy of 70.7%, 98.2%, and 93.1% were reported for [^18^F]FDG-PET/CT, PET/MRI, and MRI
Eglinton et al. [65]	2010	Prospective study	20 patients from March 2006 to September 2007 with rectal adenocarcinoma	To assess the role of [^18^F]FDG PET/CT compared to CT and MRI alone in initial staging of primary rectal adenocarcinoma	PET/CT identified primary tumor in all patients with 100% sensitivity and suggested presence of incidental extra-rectal lesions
Faneyte et al. [66]	2008	Retrospective study	70 locally recurrent rectal cancer patients between January 2003 to October 2006	To evaluate the findings of PET, CT, and MRI for lymph node metastases	PET findings differed from CT and MRI in 35% cases; 11% of the cases could have been prevented for unnecessary operations based on PET scans
Zhou et al. [67]	2021	Prospective study	56 patients with colorectal liver metastases	To evaluate simultaneous abdominal [^18^F]FDG-PET/CT and [^18^F]FDG-PET/MRI in detection of liver metastases	[^18^F]FDG-PET/MRI showed higher accuracy (99.5%) compared to [^18^F]FDG-PET/CT (47.5%), except where [^18^F] FDG-PET/CT showed a valuable result in extrahepatic disease detection
Agrawal et al. [68]	2022	Prospective study	44 rectal cancer patients	To assess [^18^F]FDG-PET/CT in metastatic spread in rectal cancer	9 patients were detected to have an extra-pelvic site of metastasis; 5 had disease upstaging after PET scan; detection of metastasis led to change in treatment plan in 7 patients

**Table 6:** Summary of key findings from studies on response assessment in rectal cancer patients.

Author	Year	Study Design	Sample Size	Study Objective	Key Findings
Ince et al. [14]	2022	Retrospective study	14 rectal cancer patients	To determine whether [^18^F]FDG-PET can give clinical complete response (cCR) over MRI alone.	[^18^F]FDG-PET provided added value in over 80% of restaging cases, based on reader assessments. [^18^F]FDG-PET/MRI assessments of cCR status at post-TNT restaging had an accuracy of 100%, compared with 71% for MRI alone
Ruggieri et al. [73]	2007	Retrospective study	136 rectal cancer patients before surgery	To evaluate downstaging in patients treated with preoperative chemoradiation	Out of 136 patients, 44 patients had complete response, 52 patients had partial response, 37 patients showed no change, and 3 patients had progression
Van Der et al. [74]	2013	Systematic review and meta-analysis		To assess diagnostic performance of MRI on restaging locally advanced rectal cancer	Restaging with DWI showed sensitivity of 83.6% and specificity of 84.8%, and a moderate result for restaging of CRM
Plodeck et al. [75]	2021	Retrospective study	40 rectal cancer patients	To assess [^18^F]FDG-PET/MRI in diagnosis of local recurrence of rectal cancer	[^18^F]FDG-PET/MRI showed sensitivity of 94%, specificity of 88%, and accuracy of 93% in detecting recurrence
Capirci et al. [77]	2007	Prospective study	44 rectal cancer patients who underwent chemoradiation therapy between December 2003 and December 2005	To assess [^18^F]FDG-PET/CT in prediction of response treatment	[^18^F]FDG-PET/CT specificity in the detection of neoadjuvant response ranged between 70% and 80%
Lambrecht et al. [78]	2010	Prospective study	22 rectal adenocarcinoma patients between May 2005 and August 2009	To assess and compare [^18^F]FDG PET with DWI-MRI for response assessment during and after CRT	In patients with pCR after CRT, [^18^F]FDG-PET showed sensitivity and specificity of 100% and 94%, with DWI-MRI showing 100% sensitivity and 87.5% specificity
Kariv et al. [79]	2025	Retrospective study	Between January 2015 and September 2022, 60 patients underwent restaging MRI; 54 underwent restaging FDG-PET; and 32 were evaluated by both	To compare [^18^F]FDG-PET and MRI in response assessment to neoadjuvant therapy	In all restaging cases, MRI interpretation was better than [^18^F]FDG-PET in terms of accuracy (78.3% vs. 68.5%), sensitivity (58.3% vs. 53.8%), and specificity (83.3% vs. 73.2%)
Sorenson et al. [80]	2019	Retrospective study	125 patients with LARC who underwent CRT, pre-CRT, and post-CRT PET-CT	To compare pre- and post-CRT PET-CT for predicting pCR in patients undergoing NAT	Post-CRT with SUV_max_ < 4.3 showed sensitivity of 65% for predicting pCR
Tey et al. [81]	2023	Prospective study	31 patients between April 2015 and August 2019 with stage I-III adenocarcinoma planned for pCR	To assess sensitivity and specificity of combined [^18^F]FDG-PET/MRI in treatment response	Combined [^18^F]FDG-PET/MRI shows primary tumor response with SUV_max_ = 11.2
Ippolito et al. [82]	2015	Prospective study	31 patients with rectal cancer	To assess ADC and SUV_max_ values with pathologic response (TRG) before and after chemoradiation therapy	Before neoadjuvant CRT, mean ADC of responders and non-responders was 0.93 and 1.03 with post-CRT ADC accuracy of 67%
Murata et al. [83]	2018	Retrospective study	44 rectal cancer patients	To determine most reliable pCR in patients who underwent preoperative chemoradiotherapy	Accuracies of predicting pCR using [^18^F]FDG-PET/CT and MRI were 78% and 61%
Aiba et al. [84]	2014	Retrospective study	40 patients with LARC	To assess value of MRI and [^18^F]FDG-PET/CT for tumor response to neoadjuvant chemotherapy (NAC)	MRI-TV2 and MRI-TV are accurate factors to assess pathological response to NAC. Addition of FDG-PET/CT to MRI did not improve performance
Asoglu et al. [85]	2020	Retrospective study	66 rectal patients	To assess [^18^F]FDG-PET/CT during total neoadjuvant treatment (TNT)	65% patients with cCR were managed nonoperatively; 35% patients underwent surgery
Avci et al. [86]	2023	Retrospective study	71 rectal cancer patients	To evaluate MRI and [^18^F]FDG-PET/CT in correct staging and predicting pathological response	57 patients underwent surgery, 84.2% reported downstaging and underwent surgery; sensitivity and specificity of MRI—91.6%, 22.2% and [^18^F]FDG-PET/CT were 100%, 12.5%
Denecke et al. [87]	2005	Retrospective study	23 rectal cancer patients	To assess CT, MRI, [^18^F]FDG-PET in prediction outcome of neoadjuvant radio chemotherapy	Histopathology showed response to neoadjuvant therapy in 13 patients whereas 10 patients were non-responders; sensitivity and specificity of [^18^F]FDG-PET-100%, 60%; CT-54%, 80% and MRI-71%, 67%
Ferri et al. [88]	2025	Prospective study	33 rectal cancer patients	To evaluate [^18^F]FDG-PET/MRI and MRI in treatment response	[^18^F]FDG-PET/MRI showed sensitivity, specificity, and accuracy of 88%, 80%, and 84% compared to MRI (82%, 50%, 67%)
Vuijk et al. [89]	2023	Prospective study	19 LARC patients	To evaluate [^18^F]FDG-PET/CT and multiparametric MRI in predicting response to NAT	26.3% were good responders; 73.7% were poor responders
Schurink et al. [90]	2021	Retrospective study	61 LARC patients	To evaluate and predict response treatment using multiparametric [^18^F]FDG-PET/CT and MRI	31 were good responders (TRG1-2); 30 poor responders (TRG3-5)

**Table 7:** Summary of key findings from studies on PET tracers in rectal cancer.

Author	Year	Study Design	Sample Size	Study Objective	Key Findings
Arulamplam et al. [94]	2003	Retrospective study	17 rectal cancer patients	To compare cellular uptake of ^18^FLT and ^18^FDG in rectal cancer patients	Out of 50 malignant lesions, 98% and 83% detected with ^18^FDG and ^18^FLT, out of 32 liver metastases, 97% visualized with ^18^FDG and 34% with ^18^FLT
Puri et al. [95]	2017	Retrospective study	11 rectal cancer patients	To compare ([^18^F]FMISO)PET scans for predicting response treatment	8 patients underwent mesorectal surgery out of which 5 were good responders. Tumor hypoxia volume was identified to have increased in 3 patients and reduced in 5 patients in week 2 of chemoradiation therapy
Wieder et al. [96]	2007	Retrospective study	10 patients with locally advanced rectal cancer	To find correlation of FLT uptake with histopathological findings	Pre-therapy FLT uptake: 4.2 ± 1.0 SUV 14 days post-chemoradiotherapy: Decreased to 2.9 ± 0.6 SUV (−28.6% ± 10.7%, *p* = 0.005) Preoperative scan: Further decreased to 1.9 ± 0.4 SUV (−54.7% ± 7.6%, *p* = 0.005)
Havelund et al. [97]	2012	Retrospective study	14 patients with locally advanced rectal cancer	To evaluate feasibility of [^18^F]FAZA PET/CT in locally advanced rectal cancer patients	[^18^F]FAZA uptake was higher in rectal tumors compared to both muscles (*p* < 0.007) or normal intestinal wall (*p* < 0.002)
Uslu et al. [98]	2021	Prospective study	20 patients between June 2016 and August 2017 diagnosed with LARC	To compare [^18^F]FDG PET/CT and DW-MRI in LARC response assessment	MRI was more promising with a sensitivity and specificity of 75% and 90.9%; [^18^F]FDG-PET/CT was efficient in differentiating responders
Zhang et al. [99]	2024	Prospective study	20 LARC patients from February 2023 to July 2023	To assess [^68^Ga]Ga-FAPI-04 PET/MRI, [^18^F]FDG PET/CT and contrast-enhanced MRI in predicting pathological response (pCR)	All the techniques could detect primary lesions. PET parameters showed that [^68^Ga]Ga-FAPI-04 performed better than [^18^F]FDG-PET/CT in distinguishing patients with pCR
